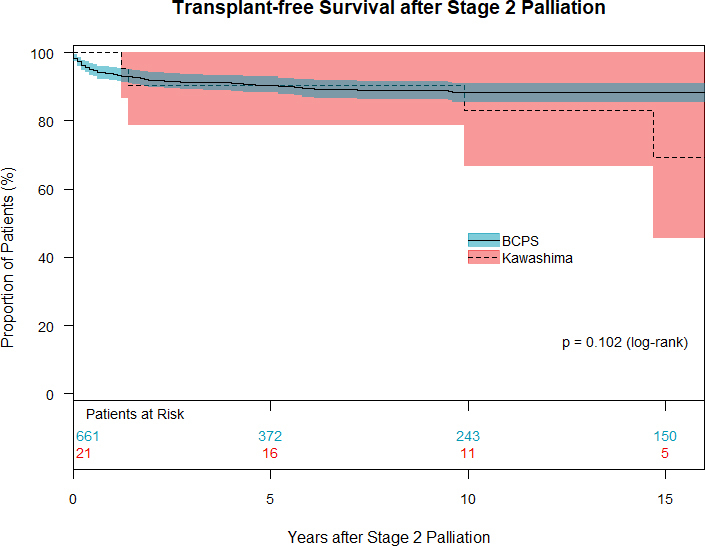# Incidence and risk factors for the development of pulmonary arteriovenous malformations after stage 2 palliation^☆^

**DOI:** 10.1016/j.ijcchd.2025.100611

**Published:** 2025-07-18

**Authors:** Lea Behrend, Thibault Schaeffer, Muneaki Matsubara, Jonas Palm, Teresa Lemmen, Nicole Piber, Paul Philipp Heinisch, Stanimir Georgiev, Alfred Hager, Peter Ewert, Jürgen Hörer, Masamichi Ono

**Affiliations:** a Department of Congenital and Pediatric Heart Surgery, German Heart Center Munich, University Hospital of Technische Universität München, Division of Congenital and Pediatric Heart Surgery, University Hospital of Munich, Ludwig-Maximilians-Universität, Europäisches Kinderherzzentrum München, Munich, Germany; b Department of Congenital Heart Disease and Pediatric Cardiology, German Heart Center Munich, University Hospital of Technische Universität München, Munich, Germany; c Department of Cardiovascular Surgery, German Heart Center Munich, University Hospital of Technische Universität München, Munich, Germany

**Keywords:** Pulmonary arteriovenous malformations, Bidirectional cavopulmonary shunt, Kawashima procedure, Total cavopulmonary connection

## Abstract

**Objective:**

This study evaluated the current incidence of pulmonary arteriovenous malformations (PAVMs) following stage 2 palliation (S2P).

**Methods:**

Patients who underwent S2P, either through a bidirectional cavopulmonary shunt (BCPS) or the Kawashima procedure (KP) between 1992 and 2022, were reviewed. The cumulative incidence of PAVMs was compared between BCPS and KP. Risk factors for the development of PAVMs were identified.

**Results:**

Among 682 patients who underwent S2P, 661 (96.9 %) underwent BCPS and 21 (3.1 %) KP. Median age at S2P was 5.1 (interquartile ranges (IQR): 3.6–9.6) months. During the median interstage follow-up of 1.6 (IQR: 1.6–2.2) years, PAVMs developed in 11 (1.6 %) patients (1.1 % (n = 7) after BCPS and 19.0 % (n = 4) after KP). Cumulative incidence of PAVMs was higher in patients after KP than those after BCPS (p < 0.001). PAVMs were observed in the right lung in 9 patients and both lungs in two. One patient with biliary atresia died of progressive PAVMs and liver cirrhosis after KP, and the remaining 10 patients underwent Fontan completion with a median interval of 1.9 (IQR: 1.5–2.4) years. PAVMs improved in all patients (9 resolutions and 1 improved). Independent risk factors for the development of PAVMs were KP (hazard ratio (HR): 16.364, p < 0.001) in all patients, anomalous pulmonary venous connection (HR: 6.772, p = 0.023) in BCPS patients, and hypoplastic left heart syndrome (HR: 18.819, p = 0.018) in KP patients.

**Conclusions:**

The incidence of PAVMs after S2P is very low after BCPS but still relevant after KP. Resolution or improvement of PAVMs is probable after Fontan completion.

## Graphical abstract

**Figure:**
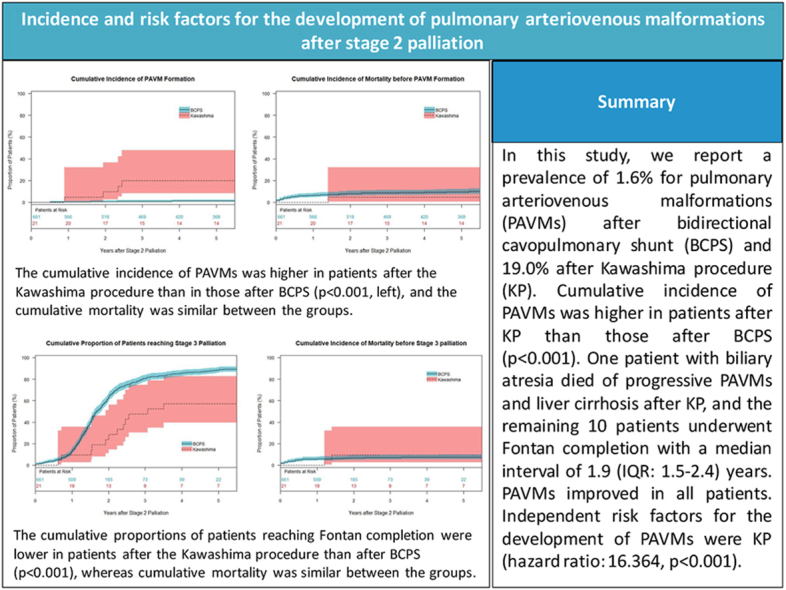

## Abbreviations and acronyms

BCPS: bidirectional cavopulmonary shunt
HLHS: hypoplastic left heart syndrome
HVI: hepatic vein incorporation
IQR: interquartile ranges
IVC: inferior vena cava
KP: Kawashima procedure
PA: pulmonary artery
PAVMs: pulmonary arteriovenous malformations
SVC: superior vena cava
TCPC: total cavopulmonary connection

## 1 Introduction

In 1958, the first successful superior vena cava (SVC) to right pulmonary artery (PA) shunt (classic Glenn shunt) was performed by William Glenn, and this procedure had been established as a palliative procedure for patients with cyanotic congenital heart disease [1,2]. Over time, the classic Glenn shunt has evolved from a unidirectional to a bidirectional cavopulmonary shunt (BCPS) as the second stage procedure for the staged Fontan palliations. The rationale of BCPS is establishing partial circulatory bypass of the right heart, providing a stable low-pressure blood flow to both lungs and volume unloading of a systemic single ventricle [3].

The development of pulmonary arteriovenous malformations (PAVMs) after the Glenn-type procedures is a serious complication [4,5]. However, the incidence of PAVMs after BCPS has become lower after the introduction of the staged Fontan strategy [6], whereas the incidence of PAVMs after the Kawashima procedure (KP) remains relevant [[7], [8], [9]].

The absence of an unidentified “hepatic factor” is a cause of the development of PAVMs [10]. Many reports have showed the resolution of PAVMs after surgical inclusion of hepatic venous flow to the lung [[11], [12], [13]]. In patients with inferior vena cava (IVC, i.e., without azygos/hemi-azygos vein continuity), the inter-stage development of PAVMs after BCPS is rare, but we identified PAVMs in some patients. Therefore, the prevention and management of PAVMs remain crucial in patients after the BCPS/KP.

In this study, we aimed to evaluate our experience in patients after stage 2 palliation, including BCPS and KP, regarding the incidence of PAVMs and the resolution of PAVMs after Fontan completion through total cavopulmonary connection (TCPC) in these subjects.

## 2 Methods

### 2.1 Data availability statement

The data that support the findings of this study are available from the corresponding author upon reasonable request.

## 3 Ethical statement

This study was approved by the Institutional Review Board of the Technical University of Munich (approval number 2024-334-S-CB on July 8, 2024). Because of the retrospective nature of the study, the need for individual patient consent was waived.

### 3.1 Patients and data collection

We reviewed all patients with a univentricular heart who underwent stage 2 palliation through BCPS or the KP from 1992 to 2022. Medical records were collected using digital and paper chart reviews, and follow-up data were collected using the institutional single ventricle database.

### 3.2 Operative techniques

BCPS was performed using cardiopulmonary bypass as described in our previous reports [14]. Cardioplegic arrest was only used for patients who required intracardiac procedures. The azygos vein was routinely divided before the initiation of cardiopulmonary bypass. The SVC was anastomosed to the right PA in an end-to-side fashion using 7-0 or 8-0 polydioxanone continuous sutures (Ethicon Inc). The KP was performed using cardiopulmonary bypass according to the original report by Kawashima et al. [15]. Patients with bilateral SVC underwent bilateral anastomoses (bilateral KP). Antegrade pulmonary blood flow was closed in most patients.

### 3.3 Diagnosis of PAVMs and measurement of PA size

PAVMs after stage 2 palliation were diagnosed when all of the following criteria were present: (1) progressive desaturation with arterial oxygen saturation in room air of 85 % or less; (2) the early appearance (within three to five cardiac cycles) of the bubble contrast in the pulmonary veins on transthoracic echocardiography; and (3) pulmonary angiography results showing the appearance of dilated distal pulmonary arteries and rapid pulmonary arterial-to-venous transit time within three heartbeats or less [10,16]. The PA index was calculated using PA angiography. The right and left PA indices were calculated by dividing the cross-sectional area of each PA branch by the body surface area. The symmetry index was calculated as described by Glatz and colleagues to evaluate the symmetric PA development [17]. The ratio of the left to the right PA index was calculated.

### 3.4 Statistical analysis

Categorical variables are presented as absolute numbers and percentages. A chi-squared test was used for categorical data. Continuous variables are expressed as medians with interquartile ranges (IQR). Levene's test was used to differentiate between normal and non-normal distributions. An independent sample Student's t-test was used to compare normally distributed variables. The Mann-Whitney *U* test was used for variables that were not normally distributed. Competing risk analysis was performed for the development of PAVMs, death, and Fontan completion. Transplant-free survival after stage 2 palliation was calculated using the Kaplan-Meier method, and the differences were determined using a log-rank test. A Cox proportional hazards model was created to identify the risk factors influencing the development of PAVMs. Data analysis was performed using SPSS version 28.0 for Windows (IBM, Ehningen, Germany) and R-statistical software (R Foundation for Statistical Computing, Vienna, Austria).

## 4 Results

### 4.1 Patient characteristics

Among 682 patients who underwent stage 2 palliation during the study period, 661 (96.9 %) patients underwent BCPS, and 21 (3.1 %) patients underwent the KP. Median age and weight at BCPS were 5.1 (interquartile ranges (IQR): 3.6–9.6) months and 5.7 (IQR: 4.8–7.1) kg, respectively. Patients' characteristics are shown in Table 1. Hypoplastic left heart syndrome (HLHS) was lower in the KP group than in the BCPS group (p = 0.024). In contrast, unbalanced atrioventricular septal defect (p = 0.011), double outlet right ventricle (DORV, p = 0.021), dextrocardia (p = 0.014), and isomerism (p < 0.001) were observed more frequently in the KP group than in the BCPS groups. Pre-BCPS/KP catheterization data are shown in Supplementary Table S1. Mean pulmonary artery pressure, transpulmonary gradient, and aortic oxygen saturation were similar between the groups. However, left atrial pressure was higher in the KP group than in the BCPS group (8 vs. 6 mmHg, p = 0.025). The right PA index was larger in the KP group than in the BCPS group (133 vs. 78 mm^2^/m^2^, p = 0.021). Perioperative data at BCPS/KP are shown in Table 2. Bilateral SVC-PA anastomosis was more frequently performed in the KP group than in the BCPS group (p < 0.001). Other variables were similar between the groups. Regarding associated liver or bile duct diseases, one patient with biliary atresia underwent the Kasai procedure before KP, and two patients had gallstones before BCPC, which were medically treated. At the time of BCPS/KP, no patient demonstrated abnormal laboratory data, including serum glutamic-oxaloacetic transaminase, glutamate pyruvate transaminase, or gamma-glutamyl transferase.

**Table 1:** Baseline characteristics of patients.

Variables	Total	BCPS	Kawashima	p-value
N (%) or median (IQR)		IVC (+)	IVC (−)	
Number of patients	682	661 (96.9)	21 (3.1)	
Primary Diagnosis				
HLHS	219 (32.1)	217 (32.8)	2 (9.5)	**0.024**
SV	128 (18.8)	116 (17.5)	12 (57.1)	**<0.001**
Tricuspid atresia	97 (14.2)	97 (14.7)	0 (0.0)	0.058
DILV	82(12.0)	81 (12.3)	1(4.8)	0.299
UAVSD	41 (6.0)	37 (5.6)	4 (19.0)	**0.011**
PAIVS	32 (4.7)	32 (4.8)	0 (0.0)	0.302
ccTGA	29 (4.3)	29 (4.4)	0 (0.0)	0.327
Others	64 (9.4)	62 (9.4)	2 (9.5)	0.982
Associated anomaly				
TGA	193 (28.3)	187 (28.3)	6 (28.6)	0.978
DORV	84 (12.3)	78 (11.8)	6 (28.6)	**0.021**
CoA	84 (12.3)	83 (12.6)	1 (4.8)	0.285
Dextrocardia	60 (8.8)	55 (8.3)	5 (23.8)	**0.014**
Isomerisms	56 (8.2)	41 (6.2)	15 (71.4)	**<0.001**
Dominant right ventricle	392 (57.5)	377 (57.0)	15 (71.4)	0.189

BCPS: bidirectional cavopulmonary shunt, IQR: interquartile range, IVC: inferior vena cava.

HLHS: hypoplastic left heart syndrome, SV: single ventricle, DILV: double inlet left ventricle.

UAVSD: unbalanced atrioventricular septal defect.

ccTGA: congenitally corrected transposition of the great arteries.

TGA: transposition of the great arteries, DORV: double outlet right ventricle.

CoA: coarctation of the aorta.

**Table 2:** Perioperative variables at Kawashima/BCPS.

Variables	Total	BCPS	Kawashima	p-value
Number of patients	682	661 (96.9)	21 (3.1)	
**Operative data**				
Type of anastomosis				
Unilateral	618 (90.6)	604 (91.7)	12 (57.1)	**<0.001**
Bilateral	64 (9.4)	55 (8.3)	9 (42.9)	
CPB time (minutes)	64 (47–92)	64 (47–92)	83 (49–98)	0.704
Need aortic cross clamp (AXC)	140(23.8)	137 (24.0)	3 (17.6)	0.547
Concomitant procedure				
PA reconstruction	216 (31.9)	213 (32.4)	3 (14.3)	0.079
AVV procedure	64 (9.4)	63 (9.6)	1 (4.8)	0.456
Aorta enlargement	218 (2.7)	18 (2.7)	0 (0.0)	0.442
DKS anastomosis	12 (1.8)	11 (1.7)	1 (4.8)	0.291
APBF open	53 (7.8)	51 (7.7)	2 (9.5)	0.761
**Postoperative data**				
ICU stay (days)	6 (3–8)	6 (3–8)	5 (4–7)	0.806
Hospital stay (days)	15 (11–24)	15 (11–24)	15 (13–28)	0.554
Re-operation	80 (13.8)	78 (13.9)	2 (10.0)	0.617
Intervention	54 (9.5)	53 (9.6)	1 (5.6)	0.561
ECMO implantation	17 (2.9)	17(3.0)	0 (0.0)	0.429
Complications				
Thrombus	34 (5.1)	33 (5.1)	1 (5.9)	0.886
Pleural effusion	33 (4.9)	32 (4.9)	1 (5.3)	0.946
Diaphragm paralysis	51 (7.7)	50 (7.8)	1 (5.3)	0.686
Arrhythmia	35 (5.3)	34 (5.3)	1 (5.9)	0.910
Chylothorax	23 (3.4)	23 (3.5)	0 (0.0)	0.404
Ascites	4 (0.6)	4 (0.46	0 (0.0)	0.732
Hospital death	26 (3.9)	26 (3.9)	0 (0.0)	0.354
**Follow-up data**				
BCPS death before Fontan	50 (7.4)	48 (7.3)	2 (9.5)	0.699
Fontan completion	585 (85.9)	570 (86.2)	16 (76.2)	0.193

BCPS: bidirectional cavopulmonary shunt, CPB: cardiopulmonary bypass, PA: pulmonary artery, AVV: atrioventricular valve, DKS: Damus-Kaye-Stansel, APBF: antegrade pulmonary blood flow, ICU: intensive care unit, ECMO: extracorporeal membrane oxygenation.

### 4.2 PAVMs after stage 2 palliation

**Fig. 1:**
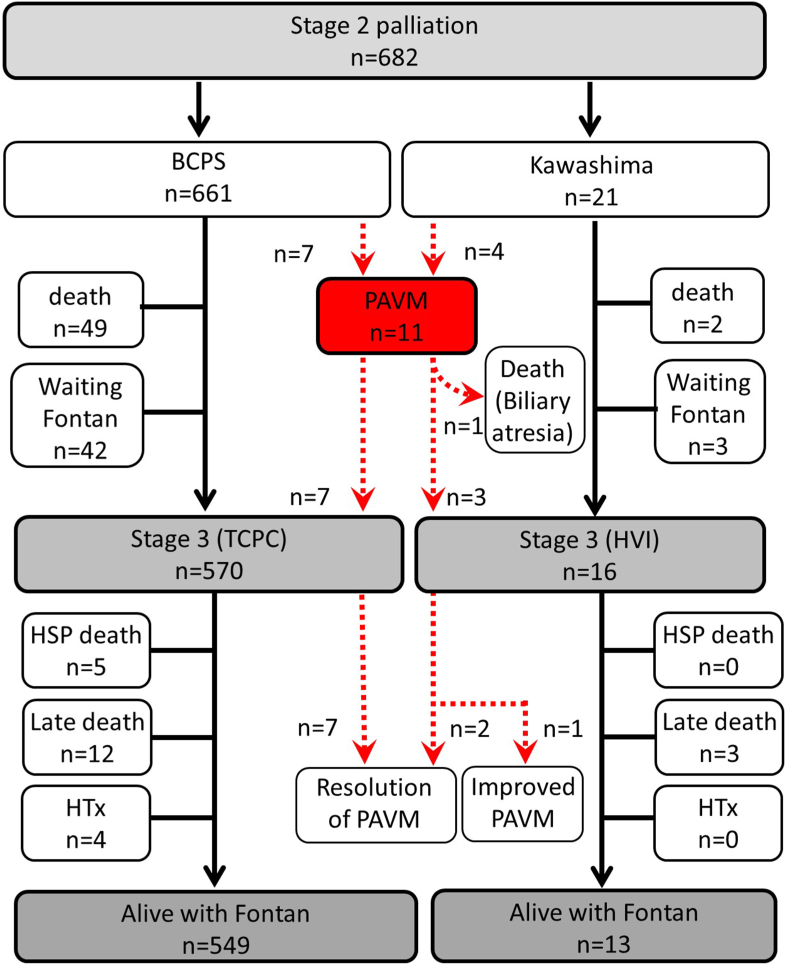
Flow chart demonstrating the details of the postoperative course after stage 2 palliation.

**Fig. 2:**
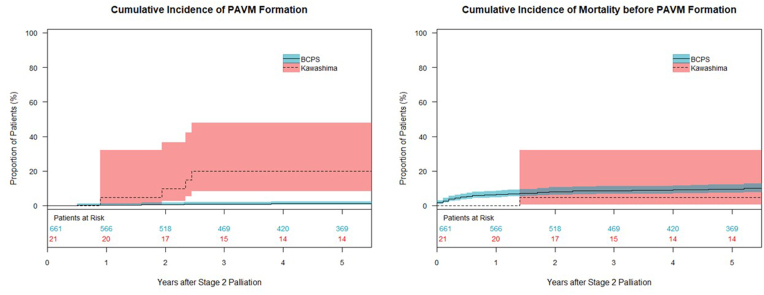
Cumulative incidence of the development of PAVMs (left) and cumulative mortality (right) for patients after BCPS (line) and those after Kawashima procedure (dot). PAVMs: pulmonary arteriovenous malformations, BCPS: bidirectional cavopulmonary shunt.

PAVMs were observed in 11 (1.6 %) patients after stage 2 palliation (7 patients (1.1 %) after BCPS and 4 patients (19.0 %) after the KP). Table 3 shows the details of 11 patients, and Fig. 1 shows the flow chart of the patients. One patient with biliary atresia developed PAVMs in both lungs 9 months after KP and died with progressive cyanosis 1.2 years postoperatively. The remaining 10 patients underwent TCPC (7 after BCPS and 3 after KP). PAVMs were observed in the right lung in 9 patients and in both lungs in 2 patients. Median oxygen saturation was 76 (IQR: 63–85) % in the pulmonary veins of the affected lung and 97 (IQR: 95–100) % in the contralateral pulmonary veins. Median oxygen saturation in the aorta was 78 (IQR: 76–81) %. The cumulative incidence of PAVMs was higher in patients after the KP than in those after BCPS (p < 0.001), whereas cumulative mortality was similar between the groups (Fig. 2). Transplant-free survival after stage 2 palliation was similar between the groups (p = 0.102, Supplementary Fig. S1). When PA indexes were compared between patients who developed PAVMs and those who did not, there were no significant differences in PA index (p = 0.504), right PA index (p = 0.194), left PA index (p = 0.536), left to right PA index ratio (p = 0.681), or PA symmetry index (p = 0.660) between the groups at the time of BCPS/KP. At the time of TCPC or hepatic vein incorporation (HVI), there were also no significant differences in PA index (p = 0.855), right PA index (p = 0.926), left PA index (p = 0.675), left to right PA index ratio (p = 0.800), or PA symmetry index (p = 0.528) between the groups.

**Table 3:** Details of patients who developed PAVMs after S2P.

Pt.	Diagnosis	S1P	S2P	Age S2P	Onset PAVMs	Affected lung	Period S2P-S3P	Age S3P	PAVMs after S3P	Course
1	DILV, TAPVC	TAPVC repair APS	BCPS	7m	1.3y	right	1.6y	2.3y	resolved	alive
2	TA Ib	(−)	BCPS	3m	2.3y	right	2.4y	2.7y	resolved	alive
3	DORV, PS, TAPVC	TAPVC repair BTTS	BCPS	14m	6m	right	0.5y	1.7y	resolved	alive
4	HLHS	Norwood BTTS	BCPS	3m	1.5y	right	1.7y	1.9y	resolved	PLE, PB, alive
5	TA Ic	PAB	BCPS	2m	1.0y	right	1.0y	1.2y	resolved	alive
6	CAVSD, PA, TGA,	BTTS	BCPS	3m	1.4y	right	1.9y	2.2y	resolved	PLE, alive
7	PAIVS, TV dysplasia	Starnes BTTS	BCPS	8m	1.9y	right	1.9y	2.6y	resolved	alive
8	HLHS	Norwood RVPAC	Kawa-shima	4m	2.5y	right	2.5y	2.8y	resolved	alive
9	DILV, PA	BTTS	Kawa-shima	6m	2y	both	2.4y	2.9y	resolved	alive
10	HLHS, TAPVC	Norwood, TAPVC repair, BTTS	Kawa-shima	18m	2.1y	right	2.0y	3.6y	improved	alive
11	DORV, MA, TAPVC, biliary atresia	BTTS	Kawa-shima	24m	1m	both		(−)	progress	liver cirrhosis, death

S1P: stage 1 palliation, S2P: stage 2 palliation, S3P: stage 3 palliation.

PAVM: pulmonary arteriovenous malformations, DILV: double inlet left ventricle.

TAPVC: total anomalous pulmonary venous connections, APS: aortopulmonary shunt.

BCPS: bidirectional cavopulmonary shunt, m: months, TA: tricuspid atresia.

DORV: double outlet right ventricle, PS: pulmonary stenosis, BTTS: Blalock-Taussig-Thomas shunt, HLHS: hypoplastic left heart syndrome, PLE: protein losing enteropathy, PB: plastic bronchitis.

PAB: pulmonary artery banding, CAVSD: complete atrioventricular septal defect.

PA: pulmonary atresia, TGA: transposition of the great arteries.

PAIVS: pulmonary atresia and intact ventricular septum, TV: tricuspid valve.

RVPAC: right ventricle to pulmonary artery conduit, MA: mitral atresia.

### 4.3 Fontan completion (TCPC or HVI) and resolution of PAVMs

**Fig. 3:**
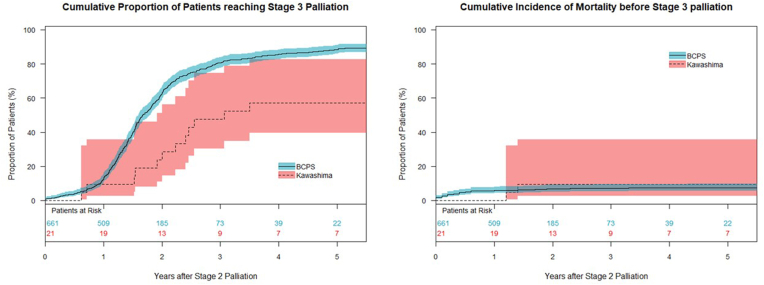
Cumulative incidence of the reaching of stage 3 Fontan completion and cumulative mortality for patients after BCPS (line) and those after Kawashima procedure (dot). BCPS: bidirectional cavopulmonary shunt.

TCPC was performed in 570 of 661 patients (86.2 %) after BCPS, and HVI was performed in 16 of 21 patients (76.2 %) after KP. The cumulative proportions of patients reaching Fontan completion were lower in patients after the KP than after BCPS (p < 0.001), whereas cumulative mortality was similar between the groups (Fig. 3). Pre-TCPC/HVI arterial oxygen saturation data are shown in Supplementary Table S2. Perioperative data are shown in Supplementary Table S3. The median age at TCPC was 2.2 (IQR: 1.8–2.9) years and the median interval between BCPS and TCPC was 1.6 (IQR: 1.8–2.9) years. The median age at HVI was 3.1(IQR: 2.7–10.5) years and the median interval between Kawashima and HVI was 2.4 (IQR: 1.5–3.5) years. Thus, Kawashima patients had a significantly older age at HVI (p < 0.001), and a longer interval between the procedures (p < 0.001) than those after BCPS. After TCPC, PAVMs improved in all patients, and median arterial oxygen saturation was 95 (IQR: 93–97) % (Fig. 1). Complete resolution of PAVMs was observed in 9 patients, and the remaining one patient demonstrated oxygen saturations of 88 % (Table 3, patient 10). Although the PAVMs were visually improved in the angiogram, the arterial oxygen saturation of this patient remained unchanged. The postoperative angiogram showed no obstruction in the TCPC pathway and no significant veno-venous collaterals. The cause of desaturation was not clearly explained.

### 4.4 Risk factors for the development of PAVMs

Risk factors for the development of PAVMs in all patients were heterotaxy (hazard ratio (HR): 6.172, p = 0.004) and KP (HR: 16.364, p < 0.001) using a univariable model (Table 4). The multivariable model revealed KP (HR: 16.364, p < 0.001) as an independent risk factor. In sub-group analysis according to the procedure, risk factors for PAVMs after BCPS were heterotaxy (HR: 6.005, p = 0.032) and anomalous pulmonary venous connection (HR: 6.772, p = 0.023) with a univariable model. The multivariable model revealed anomalous pulmonary venous connection (HR: 6.772, p = 0.023) as an independent risk factor. The risk factor for PAVMs after KP was HLHS (HR: 8.908, p = 0.030). Age at stage 2 palliation, age at stage 3 palliation, and interval between stage 2 and stage 3 palliation were not identified as a risk for developing PAVMs.

**Table 4:** Preoperative variables influencing the development of PAVMs with Cox regression model.

Variables	Univariable model			Multivariable model		
	p-value	HR	95 % CI	p-value	HR	95 % CI
**All patients**						
Age at stage 2 palliation	0.553	0.986	0.942–1.033			
Age at stage 3 palliation	0.416	0.827	0.523–1.307			
Interval between stage 2 & 3	0.567	0.850	0.488–1.482			
HLHS	0.820	0.857	0.227–3.235			
UAVSD	0.472	2.128	0.272–16.642			
Dominant RV	0.909	0.933	0.285–3.060			
Heterotaxy	**0.004**	6.172	1.804–21.087			
Dextrocardia	0.994	1.009	0.129–7.879			
Anomalous PV connection	0.100	3.681	0.780–17.370			
Kawashima procedure	**<0.001**	16.364	4.788–55.921	**<0.001**	16.364	4.788–55.921
**Patients after BCPS**						
Age at BCPS	0.459	0.949	0.825–1.091			
Age at stage 3 palliation	0.330	0.625	0.243–1.607			
Interval between stage 2 & 3	0.410	0.674	0.264–1.722			
HLHS	0.358	0.371	0.045–3.082			
UAVSD	0.190	4.140	0.496–34.566			
Dominant RV	0.500	0.597	0.134–2.672			
Heterotaxy	**0.032**	6.005	1.165–30.953			
Dextrocardia	0.582	1.812	0.218–15.054			
Anomalous PV connection	**0.023**	6.772	1.307–35.087	**0.023**	6.772	1.307–35.087
**Patients after Kawashima**						
Age at Kawashima	0.398	0.966	0.890–1.047			
Age at stage 3 palliation	0.479	0.782	0.397–1.543			
Interval Kawashima to stage 3	0.427	0.789	0.439–1.416			
HLHS	**0.018**	18.819	1.667–212.450	**0.018**	18.819	1.667–212.450
UAVSD	0.507	0.033	0.000–777.229			
Dominant RV	0.761	1.420	0.147–13.693			
Heterotaxy	0.337	0.382	0.054–2.719			
Dextrocardia	0.454	0.029	0.000–301.274			
Anomalous PV connection	0.495	0.028	0.000–823.839			

PAVMs: pulmonary arteriovenous malformations, HR: hazard ratio, CI: confidence interval.

HLHS: hypoplastic left heart syndrome, UAVSD: unbalanced atrioventricular septal defect.

RV: right ventricle, PV: pulmonary vein, BCPS: bidirectional cavopulmonary shunt.

## 5 Discussion

Among 682 patients who underwent stage 2 palliation, 11 (1.6 %) patients developed PAVMs. While only 1.1 % (n = 7) of BCPS patients demonstrated PAVMs, 19.0 % (n = 4) of subjects after KP revealed PAVMs. The cumulative incidence of PAVMs was higher in patients after KP than in those after BCPS. PAVMs resolved/improved in all patients who underwent Fontan completion. KP was an independent risk factor for PAVMs in all patients.

### 5.1 The incidence of PAVMs after BCPS and KP

The development of PAVMs after the Glenn procedure is well known after Glenn's report [18]. In the early era of 1950–1970s, the original Glenn-type procedure (SVC to right PA anastomosis) was performed as a definitive palliative procedure and the incidence of PAVMs after this procedure was reported to be 20–60 % in the long-term follow-up (10–20 years) [[19], [20], [21], [22]]. However, in the recent era, bidirectional Glenn procedure (BCPS) has been performed as stage II palliation, followed by Fontan completion within two years. As a result, the incidence of PAVMs after BCPS has decreased dramatically and has become clinically not relevant. Recent studies by Yi et al. demonstrated an incidence of 1.4 % for the development of PAVMs between BCPS and TCPC, which is consistent with our results of 1.6 % [23]. On the other hand, the incidence of PAVMs after KP remains relevant (around 20 %) since this procedure is also being performed as a second-stage palliation, followed by subsequent HVI [8,24,25]. In this study, PAVMs developed frequently in the right lung. We assume that the amount of blood flow might be one cause of the development of PAVMs. Usually, with right-sided SVC, BCPS flow mainly goes into the right lung and the right-to-left ratio is around 70 %–30 %. Therefore, we believe that the amount of blood flow through the lung might contribute to the development of PAVMs. In addition, we assume that the higher pulmonary blood flow after KP might contribute to the high incidence of PAVMs. After the Kawashima procedure, around three-quarters of the systemic venous return flows into the lungs, compared to one-half in the standard BCPS.

Many studies have reported the resolution of PAVMs after HVI and early timing of HVI is recommended, such as swift HVI 1–2 years after KP [[26], [27], [28], [29], [30]]. Early HVI also results in improved arterial oxygen saturation and regression of collateral vessels. In this study, all patients with PAVMs after the stage 2 procedure showed resolved/improved PAVMs after Fontan completion with a median duration of 1.6 years. These results are consistent with previous reports.

### 5.2 Risk factors for the development of PAVMs after stage 2 palliation

As previous studies have mentioned, KP is associated with a high rate of PAVM formation regardless of age at the procedure [[7], [8], [9],[27], [28], [29], [30]]. PAVMs can occur within a few months after KP, although the risk of PAVMs increases after several years. Heterotaxy is also one of the risk factors for the development of PAVMs [7]. The high association of portosystemic shunt with heterotaxy might be a reason or the higher volume in the pulmonary circulation could be an explanatory reason for this. In this study, most PAVMs occurred in the right lung. Usually, more than half of the pulmonary blood flow goes to the right lung, and our data showed that the left to right ratio of PA size was 0.68 before BCPS/Kawashima and 0.80 before TCPC/HVI. Therefore, the amount of blood flow might be an important factor for the development of PAVMs. However, the exact mechanisms remain unclear [31]. As the incidence of PAVMs after the Kawashima procedure is relevant, adding/leaving controlled antegrade pulmonary blood flow during the Kawashima procedure may be a good option to prevent the development of PAVMs [25].

In a sub-group analysis using only patients after BCPS (non-Kawashima patients), anomalous pulmonary venous connection was identified as a risk for the development of PAVMs. We could not find a relationship between anomalous pulmonary venous connection and PAVMs in the literature, and we believe it to be a new finding that anomalous pulmonary venous connection is a risk for PAVM in non-Kawashima patients. In this study, anomalous pulmonary venous connections were repaired before stage 2 palliation in all patients. However, residual pulmonary vein stenosis might cause congestion in pulmonary circulation, create pulmonary venous hypertension, and pose a risk for the development of PAVMs. Further studies are necessary to clarify this issue. In patients after KP, HLHS was identified as a risk factor for PAVMs. The incidence of HLHS associated with interrupted IVC seems to be low. However, Miller et al. reported in their large multi-center study of 253 KP patients that HLHS was associated with 42 (17 %) patients, and the Norwood procedure was performed in 76 (30 %) patients [29]. Unfortunately, the risk analysis for PAVMs was not performed in this study. Further studies are necessary to clarify whether HLHS is a risk for developing PAVMs after KP.

### 5.3 Future prospective

Although PAVMs were resolved after Fontan completion in patients with the current strategy of early staged Fontan palliation, the development of PAVMs remained relevant in patients who had undergone KP. In patients with anomalous pulmonary venous connection, the risk of PAVMs was higher after BCPS. Therefore, maintaining controlled antegrade pulmonary blood flow at stage 2 palliation may be a good option to prevent the development of PAVMs. The disadvantage of this option is an additional volume load for the systemic ventricle and a possible increase in the degree of atrioventricular valve regurgitation [32,33]. However, maintaining only a small amount of antegrde pulmonary blood flow could be sufficient to prevent the development of PAVMs. The indication for this option should be discussed and performed carefully in individual patients.

### 5.4 Limitations

Limitations are its retrospective nature and single-center analysis. Surgical and medical management may have changed during the study period, probably influencing the long-term outcomes. As this study focused on a rare clinical entity, the limited number of patients does not allow us to capture clinical differences between subgroups, and the statistical inferences should be treated with caution. Small PAVMs might be missed using this clinical diagnosis.

## 6 Conclusions

In the staged Fontan palliation approach, the incidence of PAVMs is low following BCPS but remains clinically significant in patients following KP. While the development of PAVMs is a concern in this subset of patients, the majority experience resolution after the completion of the Fontan procedure, highlighting the effectiveness of the final stage in addressing this complication.

## CRediT authorship contribution statement

**Lea Behrend:** Writing – original draft, Data curation. **Thibault Schaeffer:** Writing – original draft, Visualization, Formal analysis. **Muneaki Matsubara:** Writing – original draft, Visualization, Formal analysis, Data curation. **Jonas Palm:** Writing – original draft, Investigation, Formal analysis, Data curation. **Teresa Lemmen:** Writing – original draft, Validation, Formal analysis, Data curation. **Nicole Piber:** Writing – original draft, Investigation, Formal analysis, Data curation. **Paul Philipp Heinisch:** Writing – review & editing, Investigation, Formal analysis. **Stanimir Georgiev:** Writing – review & editing, Validation, Supervision, Methodology, Investigation, Data curation. **Alfred Hager:** Writing – review & editing, Validation, Supervision, Project administration, Methodology, Conceptualization. **Peter Ewert:** Writing – review & editing, Validation, Supervision. **Jürgen Hörer:** Writing – review & editing, Validation, Supervision, Project administration, Funding acquisition, Conceptualization. **Masamichi Ono:** Writing – original draft, Visualization, Formal analysis, Data curation, Conceptualization.

## Funding statement

This study was supported by grants from the Förderverein des Deutschen Herzzentrums München.

## Declaration of competing interest

The authors declare no potential conflicts of interest with respect to the research, authorship, or publication of this article.

## Figures and Tables

**figs1:**