# Supplementary material for: Incidence and risk factors for the development of pulmonary arteriovenous malformations after stage 2 palliation
Source: Int J Cardiol Congenit Heart Dis. 2025 Jul 18;21:100611. doi: 10.1016/j.ijcchd.2025.100611 (PMC12457743; doi:10.1016/j.ijcchd.2025.100611)
Supplement: Multimedia component 1 [file mmc1.docx]

**Supplementary Tables**

**Supplementary Table S1**

| Table S1. Pre Kawashima/BCPS catheterization data | | | |  |  |
| --- | --- | --- | --- | --- | --- |
| Variables | | Total | BCPS | Kawashima | P-value |
| **Catheterization data** | |  |  |  |  |
|  | Hemoglobin (g/dL) | 14.0 (12.4-15.4) | 13.9 (12.3-15.4) | 14.8 (13.4-17.3) | 0.108 |
|  | Pulmonary artery pressure (mm Hg) | 14 (11-18) | 14 (12-17) | 13 (10-19) | 0.540 |
|  | Left atrial pressure (mm Hg) | 6 (5-8) | 6 (5-8) | 8 (6-10) | **0.025** |
|  | Transpulmonary gradient (mm Hg) | 8 (5-10) | 8 (5-10) | 4 (3-12) | 0.236 |
|  | Systolic ventricular pressure (mm Hg) | 79 (72-87) | 79 (72-87) | 77 (68-95) | 0.934 |
|  | Ventricular endo-diastolic pressure (mm Hg) | 9 (7-11) | 9 (7-11) | 10 (7-10) | 0.907 |
|  | Aortic oxygen saturation (%) | 76 (72-81) | 76 (72-81) | 75 (71-84) | 0.992 |
| Pulmonary artery index (PAI) | |  |  |  |  |
|  | PAI (mm^2^/m^2^) | 151 (116-213) | 150 (115-211) | 190 (170-295) | 0.058 |
|  | Right PAI (mm^2^/m^2^) | 80 (59-119) | 78 (58-117) | 133 (95-202) | 0.021 |
|  | Left PAI (mm^2^/m^2^) | 67 (48-93) | 67 (47-92) | 83 (57-112) | 0.530 |
|  | Left to right ratio | 0.83 (0.57-1.07) | 0.84 (0.58-1.08) | 0.49 (0.43-0.85) | 0.266 |
|  | Symmetry index | 0.71 (0.52-0.88) | 0.72 (0.53-0.88) | 0.49 (0.43-0.79) | 0.496 |
| N (%) or median (IQR) | |  |  |  |  |

**Supplementary Table S2**

| Supplementary Table S2. Pre TCPC/HVI arterial oxygen data and pulmonary artery size | | | | |  |
| --- | --- | --- | --- | --- | --- |
| Variables | | Total | TCPC | HVI | P-value |
| Number of patients | | 586 | 570 | 16 |  |
| Pre-TCPC/HVI PAVM (+) | | 10 (1.7) | 7 (1.2) | 3 (18.8) | <0.001 |
| Catheterization data | |  |  |  |  |
|  | Aortic oxygen saturation (%) | 83 (80-86) | 83 (80-86) | 83 (81-84) | 0.746 |
|  | PAVM (-) | 83 (80-86) | 83 (80-86) | 83 (82-86) | 0.640 |
|  | PAVM (+) | 78 (77-81)* | 78 (75-78)* | 81, 81, and 82 | 0.013 |
| Pulmonary artery index (PAI) | |  |  |  |  |
|  | PAI (mm^2^/m^2^) | 165 (132-216) | 166 (131-216) | 163 (155-197) | 0.928 |
|  | Right PAI (mm^2^/m^2^) | 105 (77-137) | 104 (77-138) | 119 (98-124) | 0.812 |
|  | Left PAI (mm^2^/m^2^) | 60 (43-87) | 60 (43-87) | 64 (38-79) | 0.742 |
|  | Left to right ratio | 0.59 (0.39-0.85) | 0.59 (0.39-0.86) | 0.63 (0.32-0.75) | 0.455 |
|  | Symmetry index | 0.58 (0.39-0.77) | 0.57 (0.39-0.77) | 0.63 (0.32-0.75) | 0.982 |
| N (%) or median (IQR) | |  |  |  |  |
| *: p<0.05 between PAVM (-) vs. PAVM (+) | | |  |  |  |

**Supplementary Table S3**

| Table S3. Perioperative data at TCPC/hepatic vein incorporation | | | |  |  |
| --- | --- | --- | --- | --- | --- |
| Variables | | Total | TCPC | HVI | P-value |
| Number of patients | | 586 | 570 (97.3) | 16 (2.7) |  |
| Age at TCPC/HVI | | 2.2 (1.8-3.0) | 2.2 (1.8-2.9) | 3.1 (2.7-10.5) | <0.001 |
| Interval stage II to III | | 1.6 (1.3-2.2) | 1.6 (1.2-2.2) | 2.4 (1.5-3.5) | <0.001 |
| EC-TCPC | | 561 (95.7) | 545 (95.6) | 16 (100.0) | 0.392 |
| Conduit diameter | |  |  |  |  |
|  | 14 | 1 (0.2) | 0 (0.0) | 1 (6.3) | <0.001 |
|  | 16 | 7 (1.2) | 4 (0.7) | 3 (18.8) |  |
|  | 18 | 495 (88.2) | 486 (89.2) | 9 (56.3) |  |
|  | 20 | 48 (8.6) | 46 (8.4) | 2 (12.5) |  |
|  | 22 | 10 (1.8) | 9 (1.7) | 1 (6.3) |  |
| CPB time (minutes) | | 64 (46-92) | 64 (47-91) | 56 (41-105) | 0.898 |
| Aortic cross clamp (AXC) | | 131 (22.4) | 127 (22.3) | 4 (26.7) | 0.688 |
| Fenestration | | 37 (6.3) | 36 (6.3) | 1 (6.7) | 0.956 |
| Concomitant procedure | |  |  |  |  |
|  | PA reconstruction | 50 (8.5) | 50 (8.8) | 0 (0.0) | 0.215 |
|  | AVV procedure | 74 (12.6) | 70 (12.3) | 4 (25.0) | 0.131 |
|  | Atrioseptectomy | 21 (3.6) | 21 (3.7) | 0 (0.0) | 0.434 |
|  | DKS | 10 (1.7) | 10 (1.8) | 0 (0.0) | 0.593 |
| **Postoperative data** | |  |  |  |  |
| ICU stay (days) | | 6 (4-8) | 6 (4-8) | 12 (7-16) | 0.120 |
| Hospital stay (days) | | 20 (14-27) | 20 (14-26) | 23 (20-39) | 0.110 |
| Complications | |  |  |  |  |
|  | Pleural effusion | 290 (49.8) | 279 (49.2) | 11 (73.3) | 0.065 |
|  | Chylothorax | 132 (22.7) | 127 (22.4) | 5 (33.3) | 0.320 |
|  | Ascites | 113 (19.4) | 107 (18.8) | 6 (40.0) | 0.041 |
| Hospital death | | 5 (0.9) | 5 (0.9) | 0 (0.0) | 0.707 |
